# Clinical-histopathological features and cancer gene analysis of cutaneous epithelioid angiosarcoma: A report of 4 cases

**DOI:** 10.1016/j.jdcr.2023.11.039

**Published:** 2024-02-10

**Authors:** Keiko Tokuchi, Teruki Yanagi, Suguru Kurosawa, Shinya Kitamura, Takuya Maeda, Che Yuan Hsu, Kodai Miyamoto, Hiroshi Nishihara, Hideyuki Ujiie

**Affiliations:** a Department of Dermatology, Faculty of Medicine and Graduate School of Medicine, Hokkaido University, Sapporo, Japan; b Genomics Unit, Keio Cancer Center, Keio University School of Medicine, Tokyo, Japan

**Keywords:** cancer genome analysis, epithelioid angiosarcoma, nodule, plaque, purpura

## Introduction

Angiosarcoma (AS) is a rare variant of malignant soft tissue sarcoma, representing less than 1% of all soft tissue sarcomas.^1^ Among the histopathological subtypes of AS, epithelioid angiosarcoma (EAS) is characterized by epithelioid morphology, with polygonal or spherical cells containing eosinophilic cytoplasm.^2^ Tumors where over 80% of the cells are epithelioid are typically classified as EAS.^3^ EAS usually manifests during adulthood, particularly around the seventh decade of life.^2^ Approximately 12% of cutaneous AS is cutaneous EAS, yet detailed studies on clinical presentations are lacking.^2^ In this study, we report 4 cases of EAS and analyze the vascular space in biopsy specimens to investigate correlations between clinical and histopathological findings. Additionally, we conducted cancer gene analyses using two EAS cases and three AS cases, and we detected no notable genetic differences between EAS and conventional AS tumors.

## Case report

**Fig 1:**
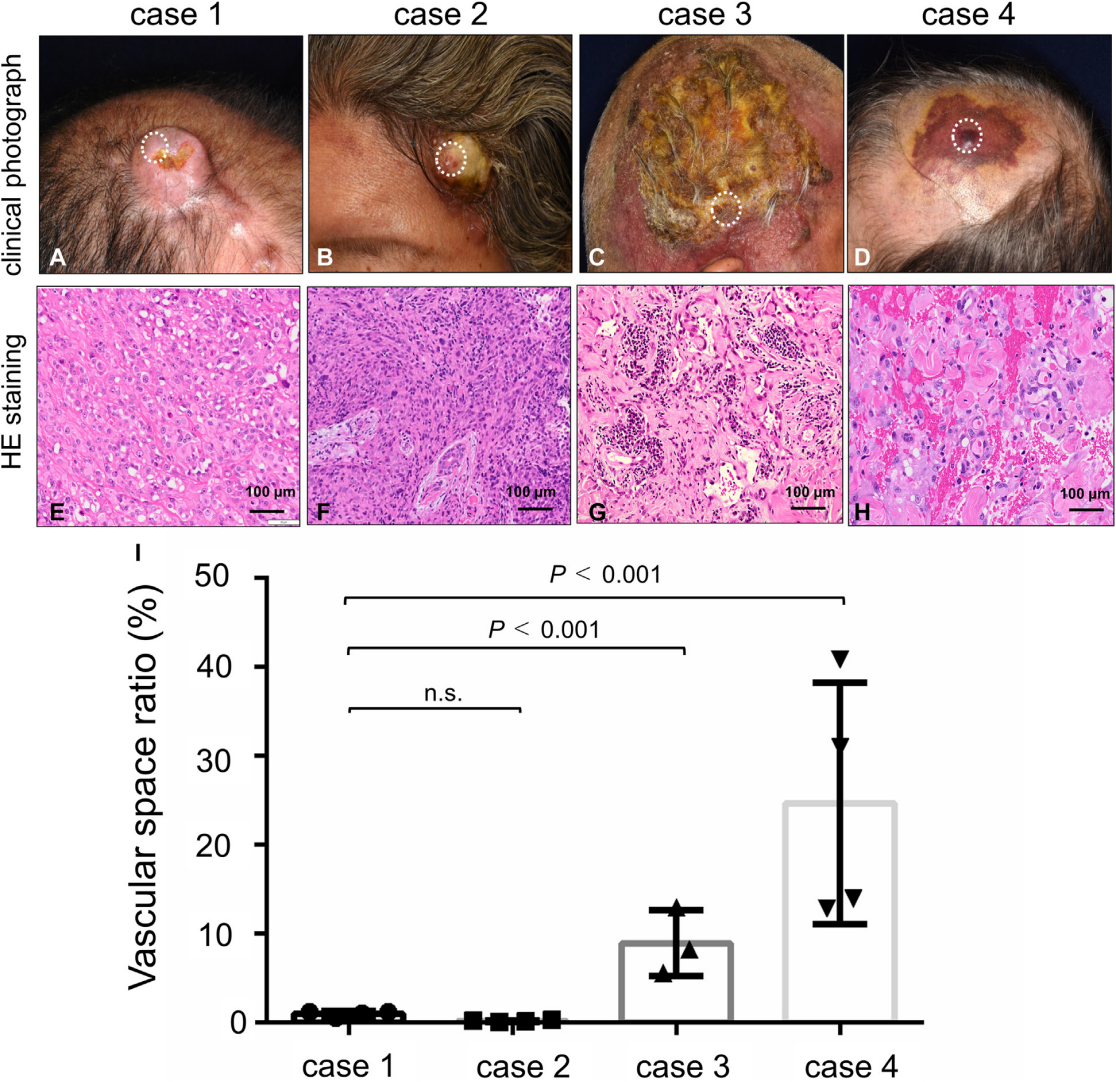
Clinical and histopathological findings of the present 4 cutaneous epithelioid angiosarcomas. **A** to **D,** Clinical photographs of the present cases. *Dotted circles* indicate biopsy sites. **E** to **H,** Histopathological findings. Scale bar = 100 μm. Hematoxylin and eosin staining. **I,** A bar graph indicates the vascular space ratio for each case. These ratios were calculated using Image J software. We photographed high-power view images randomly and colored vascular channels manually to calculate the vascular space ratio (*n* = 3 or 4, mean ± SD, *t* test).

We report 4 individuals diagnosed with EAS (Supplementary Table I, available via Mendeley at https://doi.org/10.17632/rvs83cnfph.1; Fig 1, *A*-*D*). In all cases, skin biopsies revealed a solid sheet of polygonal or round tumor cells with eosinophilic cytoplasm and intracytoplasmic lumina formations (Fig 1, *E*-*H*). Immunohistochemically, the tumor cells in all cases were positive for CD31 but negative for smooth muscle actin, S-100, Melan-A, and HMB-45 (not shown). Consequently, all 4 cases were diagnosed as cutaneous EAS. Purpura was not observed in the lesions of 2 of the 4 cases (cases 1 and 2), whereas purpura was observed in the others (cases 3 and 4). To analyze the correlation between clinical manifestations and histopathological findings, we examined the histopathological vascular space in the skin biopsy specimens. The vascular space ratio, defined as the ratio of the vascularized channel area to the total high-power view area, was calculated using Image J software (https://imagej.nih.gov/ij/).^4^ The vascular space ratio was 1.10% for case 1, 0.19% for case 2, 8.95% for case 3, and 24.67% for case 4, indicating that tumors with a high amount of vascular space tend to display purpura (Fig 1, *I*). For the genomic analyses of the EAS tumors, we performed somatic gene mutation analysis of cancer-associated genes for 2 of the EAS cases (case 1 and case 3) and for 2 conventional AS cases (both 81-year-old males), as well as for 1 case of Stewart-Treves syndrome (lymphedema-associated AS, an 89-year-old female) (Table I, Supplementary Table II, available via Mendeley at xxx, Institutional Review Board approval #15-029). In both EAS cases, loss-of-function mutations in tumor protein 53 (*TP53*) were detected. Additionally, loss of heterozygosity of cyclin-dependent kinase inhibitor 2A (case #1) and amplification of guanine nucleotide-binding protein alpha subunit (case #3) were observed. One of the 2 conventional AS cases showed a v-myc avian myelocytomatosis viral related oncogene, neuroblastoma derived homolog mutation (ClinVar: likely pathogenic), and the other case showed loss of heterozygosity of cyclin-dependent kinase inhibitor 2A and *TP53* with a high tumor mutation burden (11 mutants/Mbp). The case with Stewart-Treves syndrome exhibited amplification of the *MYC* oncogene, consistent with previous studies.^5^ The data suggests that gene mutations in EAS resemble those in AS, including loss-of-function mutations of tumor suppressor genes (*TP53* and cyclin-dependent kinase inhibitor 2A).

**Table I:** Actionable mutations of epithelioid angiosarcoma, conventional angiosarcoma, Stewart-Treves syndrome

Epithelioid AS #1				
Gene	AA Change	SAF	CNA	eCN
*TP53*	p.Y220C	0.31707317	HD	−0.3
*KEAP1*	Wild type		HD	−0.2
*TP53*	Wild type		HD	−0.3
*TSC1*	Wild type		HD	−0.2
*PMS2*	Wild type		LOH	0.2
*BRCA1*	Wild type		LOH	0.5
*BRCA2*	Wild type		LOH	0.7
*CHEK2*	Wild type		LOH	0.5
*PALB2*	Wild type		LOH	0.5
*CDKN2A*	Wild type		LOH	0.9
*SMARCB1*	Wild type		LOH	0.8
*POLD1*	Wild type		LOH	0.7
*STK11*	Wild type		LOH	0.9
*VHL*	Wild type		LOH	0.3
*FANCA*	Wild type		LOH	0.5
*CDH1*	Wild type		LOH	0.6
*EP300*	Wild type		LOH	0.6
*SMARCA4*	Wild type		LOH	0.4
*PIK3R2*	Wild type		LOH	0.9
*EPCAM*	Wild type		LOH	0.2
*RAD51C*	Wild type		LOH	0.2

Epithelioid AS #3				
Gene	AA Change	SAF	CNA	eCN
*GNAS*	Wild type		Amplification	14.8
*EZH2*	Wild type		Amplification	4.5
*BRAF*	Wild type		Amplification	4.5
*MYC*	Wild type		Amplification	4
*TP53*	c.376-1G > A	0.55555556	LOH	1.1
*BRCA2*	Wild type		LOH	1.3
*TP53*	Wild type		LOH	1.1
*MAP3K1*	Wild type		LOH	1.4
*RB1*	Wild type		LOH	1.4
*APC*	Wild type		LOH	1.4
*FBXW7*	Wild type		LOH	1.4
*BCL2L11*	Wild type		LOH	1.4

Conventional AS #1 (TMB-high, 11 Muts/Mbp)				
Gene	AA Change	SAF	CNA	eCN
*FGFR4*	Amplification		Amplification	4.5
*CDKN2A*	Wild type		LOH	0.6
*TP53*	Wild type		LOH	1.1
*VHL*	Wild type		LOH	1.1
*KEAP1*	Wild type		LOH	0.9

Conventional AS #2				
Gene	AA Change	SAF	CNA	eCN
*MYCN*	p.P44L	0.206198	Neutral	0.8

Stewert Treves syndrome				
Gene	AA Change	SAF	CNA	eCN
*MYC*	Amplification		Amplification	101.3
*NF1*	p.V1182F	0.340458	LOH	1.1
*PIK3CA*	p.Q546K	0.080696	neutral	2.4
*PALB2*	Wild type		LOH	1
*KEAP1*	Wild type		LOH	0.6
*NF1*	Wild type		LOH	1.1
*RAD51C*	Wild type		LOH	1.2

*AA*, Amino acid; *APC*, adenomatous polyposis coli; *AS*, angiosarcoma; *BCL2L11*, B-cell/CLL lymphoma 2-like 11; *BRAF*, v-raf murine sarcoma viral oncogene homolog B1; *BRCA1*, Breast cancer susceptibility gene 1; *BRCA2*, Breast cancer susceptibility gene 2; *CDH1*, cadherin 1; *CDKN2A*, cyclin dependent kinase inhibitor 2A; *CDKN2A*, cyclin-dependent kinase inhibitor 2A; *CHEK2*, checkpoint kinase2; *CNA*, copy number alteration; *eCN*, estimated copy number in cancer cells; *EP300*, E1A binding protein P300; *EPCAM*, epithelial cell adhesion molecule; *EZH2*, enhancer Of zeste 2 polycomb repressive complex 2 subunit; *FANCA*, Fanconi anemia complementation group A; *FBXW7*, F-box and WD repeat domain containing 7; *FGFR4*, fibroblast growth factor receptor 4; *GNAS*, guanine nucleotide-binding protein alpha subunit; *HD*, homo deletion; *KEAP1*, kelch like ECH associated protein 1; *KEAP1*, Kelch-like ECH-associated protein1; *LOH*, loss of heterozygosity; *MAP3K1*, mitogen-activated protein kinase kinase kinase 1; *MYC*, myelocytomatosis viral related oncogene; *NF1*, neurofibromin 1; *PALB2*, partner and localiser of BRCA 2; *PALB2*, partner and localizer of BRCA2; *PIK3CA*, phosphatidylinositol-4,5-bisphosphate 3-kinase catalytic subunit alpha; *PIK3R2*, phosphoinositide-3-kinase regulatory subunit 2; *PMS2*, post-meiotic segregation 2; *POLD1*, DNA polymerase delta1 catalytic subunit; *RAD51C*, RAD51 paralog C; *RB1*, retinoblastoma gene1; *SAF*, somatic allele frequency; *SMARCA4*, SWI/SNF-related, matrix-associated, actin-dependent regulators of chromatin A4; *SMARCB1*, SWI/SNF related, matrix associated, actin dependent regulator of chromatin, subfamily b, member 1; *STK11*, serine/threonine kinase 11; *TMB*, tumor mutation burden; *TP53*, tumor protein 53; *TSC1*, Tuberous sclerosis complex1; *VHL*, Von Hippel-Lindau tumor suppressor.

## Discussion

Rosai et al first described the epithelioid phenotype of AS in 1976, with Fletcher et al establishing the current definition in 1991.^6^ Due to the diversity of primary sites and the incredibly aggressive nature of the tumors, only around 50 reports of cutaneous EAS are publicly available. Nodules and plaques are the most common clinical manifestations of cutaneous EAS.^2^^,^^7^ In our study, half of the EAS cases presented with nonpurpuric lesions, and all had elevated nodules or plaques on the scalp. Consistent with earlier studies, our cases displayed large polygonal epithelioid cells with eosinophilic cytoplasm. EAS tumors exhibit higher cellularity of the epithelioid tumor cells, which contributes to their unique clinical presentations, such as nodule, plaque, or tumor formation. Our findings suggest that some EAS lesions exhibit purpura due to the presence of certain vascular formations.

Several mutation analysis studies on human AS have been reported.^5^^,^^8^ Gene alterations of *TP53* have been frequently reported^5^^,^^9^; thus, our cancer gene analysis results align with these previous findings. No reports on EAS-specific gene mutations exist; however, our study suggests no notable differences in cancer-associated gene mutations between EAS and conventional AS tumors.

In conclusion, we reported 4 cases of cutaneous EAS and analyzed their clinical characteristics and histopathological findings. Our study suggests that the purpuric appearance of EAS may be dependent on the vascular space formations in the EAS tumor tissue. Furthermore, our analysis of cancer-associated gene mutations indicates that EAS tumors have gene mutation profiles similar to those of conventional AS, including loss-of-function mutations in *TP53*.

## Conflicts of interest

None disclosed.
